# Whole genome data sequence for VIT_AJPSC phage against *Shigella dysenteriae*

**DOI:** 10.1186/s13104-025-07261-9

**Published:** 2025-07-01

**Authors:** Aishwarya Srivastava, N. S. Jayaprakash

**Affiliations:** Center for Bio-separation Technology, Vellore Institute of Technology, Vellore, 632014 Tamil Nadu India

**Keywords:** Antibiotic resistance, Genome, Bacteriophage, Myoviridae, *Shigella dysenteriae*

## Abstract

**Objective:**

Antibiotic resistance is one of the major causes of the increase in bacterial infections worldwilde. This is why it is the need of an hour to look for an alternative treatment; bacteriophage therapy is one of the most potent. Phages have been and still are being isolated against *Shigella species* but many of them remain uncultured and uncharacterized. Our study focuses on whole genome data sequence for bacteriophage isolated against *Shigella dysenteriae*.

**Data description:**

Bacteriophage VIT_AJPSC was isolated from cow shed drainage sample against *Shigella dysenteriae*. Phage genome is linear, double stranded and 38,898 bp long. A total 38 Coding sequences (CDS) were annotated and GC content was found to be 57%. TEM analysis showed that phage VIT_AJPSC has a icosahedral-shaped head, a contractile neck and tail region, a characteristic feature of Myoviridae family.

## Objective

There are presently four species of shigella, namely *flexneri*,* dysenteriae*,* boydii and sonnei*, that are responsible for causing shigellosis at different levels. Shigellosis caused by *Shigella dysenteriae* is contagious and life-threatening as it releases Shiga toxin that has cytotoxic effect on kidneys and central nervous system [1]. According to the latest Center for Disease Control and Prevention (CDC) report, shigellosis caused by *Shigella dysenteriae* has increased by 5% from 2015 to 2022 [2]. The probable reasons are increased antibiotic resistance and serotype conversion by *Shigella dysenteriae* type 1. Serotype conversion is a phenomenon where the pathogen changes the surface properties of target cell once it enters in it. Due to this, the usual antibiotics are not able to act upon.

This is why, there is a need to look for an alternative treatment to current antibiotics. Bacteriophages, or simply phages, are the virus that infect bacteria. The major advantage of phage over antibiotics is that it is host-specific, therefore, it will not harm the other neighbouring bacteria [3]. Phages have two components- genetic material and capsid protein. They use host machinery to replicate and are practically dead outside the host. The first phage was discovered by Felix d’Herelle in 1917 to treat shigellosis. He took faecal samples of infected patients, isolated and cultured bacteriophage from it, and used it to cure shigellosis in children [4]. He observed that phages are able to show similar results as antibiotics in the early stage of infection. After his discovery, phage therapy was tested and used to cure infectious diseases and also for bio-typing assays. In this study, we have isolated phage (VIT_AJPSC) against *Shigella dysenteriae.* The study provides insights into morphological and genomic characterization of isolated phage.

## Data description

Bacteriophage was isolated from the cow shed drainage sample collected from Tamil Nadu Veterinary and Animal Science University, Chennai. Luria broth Agar was used for the subculturing of *Shigella dysenteriae* (isolated from soil sample and identified by 16 S rRNA sequencing) to obtain pure colonies.

Pooled samples were taken and screened for the isolation of bacteriophage against *Shigella dysenteriae* via double-layer agar assay. The isolated phage was labelled as VIT_AJPSC. It was maintained at optimized temperature and pH in Sodium-Magnesium (SM) buffer. Purification and characterization (pfu, burst size, one-step growth curve) of isolated phage were done. Further, phage DNA isolation was done using the phenol-chloroform method.

DNA was quantified by QUBIT3 fluorometer [5, 6]. The initial concentration of DNA used was 100 ng. DNA was enzymatically fragmented into 200-300 bp size fragments. Fragments were converted from overhangs to blunt ends by end repair. Exonuclease activity from 3’ to 5’ end repair removes 3’overhangs and its polymerase activity fills in the 5’ overhangs. Adenylation was done by adding a single ‘A’ nucleotide to the 3’ ends of the blunt-ended fragments. Loop adapters were ligated and cleaved with the help of uracil-specific excision reagent (USER) enzyme to the adenylated fragments [7]. The samples were further purified using AMPure beads and DNA was then enriched by 6 PCR cycles using NEBNext Ultra II Q5 master mix, illumina universal primer and sample-specific octamer primers [8]. The amplified products were cleaned-upusing AMpure beads and the final DNA library was eluted in 15 µl of 0.1X TE buffer. 1 µl of the library was used to quantify by QUBIT 3 Fluorometer using dS DNA HS reagent. The fragment analysis was performed on Agilent 2100 Bioanalyzer, by loading 1 µl of the library into to Agilent DNA 7500 chip [9]. The result was a high-quality, gap-free single contig of 38,898 base pairs, representing the complete genome of the bacteriophage.

The assembled genome of bacteriophage VIT-AJPSC was annotated with Pharokka tool (source code: pharokka.py -i assembly.fasta -o pharokka) and verified by NCBI blast. Open reading frames were determined by the ORF finding tool from NCBI. Each CDS was confirmed by BLASTn tool and each protein was determined for its function by BLASTp, including terminase small and large subunit protein (CDS_0001 and CDS _0002), tail proteins (CDS_0008, CDS_0010, CDS_0013, CDS_0014, CDS_0015, CDS_0016, CDS_0023, CDS_0024, CDS_0025 and CDS_0028), lysins (CDS_0066, CDS_0067, CDS_0068, CDS_0069 and CDS_0070) and hypothetical proteins [10].

The terminase large subunit gene was used to check the evolutionary pattern and it was observed that the phage gene has close resemblance with Shigella phage Sf1V. The genomic size of isolated phage was 38,898 bp, data file 1 (Table 1) [11]. Total number of genes were 71, out of which 28 were coding sequences for functional proteins [10]. Table 2 defines the primary role of some of the functional proteins in phage genome.

**Table 1:** Overview of data files and data sets

Label	Name of data file/data set	File types (file extension)	Data repository and identifier (DOI or accession number)
Data file 1	Gene assembly	GenBank file (.gbk)	NCBI nucleotide, Accession number 0R799564 http://identifiers.org/insdc.gca:GCA_033952015.1 [11]
Data set 1	Whole genome sequence	GenBank file (.gbk)	NCBI nucleotide, Accession number 0R799564 http://identifiers.org/insdc:OR799564.1 [10]

**Table 2:** Major proteins and their primary role

Protein groups	CDS tags	Primary role
Terminase	CDS_001, CDS_002	DNA recognition, cleavage, and packaging into capsid
Tail proteins	CDS_008–28 (Selected)	Host attachment, DNA delivery, structural assembly
Lysins	CDS_0066–70	Bacterial cell wall degradation for phage release
Major head protein	CDS_006	Head and packaging
Membrane proteins	CDS_003	Moron, auxiliary metabolic gene and host takeover
Glycotransferase	CDS_0030	Moron, auxiliary metabolic gene and host takeover
Endonuclease	CDS_0057, CDS_0071	DNA, RNA and nucleotide metabolism
Transposase	CDS_0061	Integration and excision
Anti-termination protein	CDS_0058	Transcription regulation

The GC content was found to be 57% [11]. The whole genome sequence was published in NCBI and the accession number is OR799564, data set 1 [10].

## Data limitations

The study has been done in laboratory conditions and this data is limited to the VIT_AJPSC phage against *Shigella dysenteriae*. The isolated phage is very specific to the host and does not cause lysis to any other species tested. In future, studies will be conducted for the application of VIT_AJPSC phage in food such as lettuce, greens and dairy products contaminated with shigella.

## Data Availability

All the data is provided within the manuscript.

## Abbreviations

BLAST: Basic local alignment search tool
CDC: Centers for Disease Control and Prevention
CDS: Coding sequences
DNA: Deoxyribonucleic acid
PCR: Polymerase chain reaction
SM: Sodium magnesium
TEM: Transmission electron microscopy
USER: Uracil–specific excision reagent
